# Spatial distribution of Na^+^-K^+^-ATPase in dendritic spines dissected by nanoscale superresolution STED microscopy

**DOI:** 10.1186/1471-2202-12-16

**Published:** 2011-01-27

**Authors:** Hans Blom, Daniel Rönnlund, Lena Scott, Zuzana Spicarova, Jerker Widengren, Alexander Bondar, Anita Aperia, Hjalmar Brismar

**Affiliations:** 1 Department of Applied Physics, Royal Institute of Technology, Stockholm, Sweden; 2 Science for Life Laboratory, Stockholm, Sweden; 3 Department of Women's and Children's Health, Karolinska Institutet, Stockholm, Sweden; 4 Institute of Chemical Biology and Fundamental Medicine, Novosibirsk, Russia

## Abstract

**Background:**

The Na^+^,K^+^-ATPase plays an important role for ion homeostasis in virtually all mammalian cells, including neurons. Despite this, there is as yet little known about the isoform specific distribution in neurons.

**Results:**

With help of superresolving stimulated emission depletion microscopy the spatial distribution of Na^+^,K^+^-ATPase in dendritic spines of cultured striatum neurons have been dissected. The found compartmentalized distribution provides a strong evidence for the confinement of neuronal Na^+^,K^+^-ATPase (α3 isoform) in the postsynaptic region of the spine.

**Conclusions:**

A compartmentalized distribution may have implications for the generation of local sodium gradients within the spine and for the structural and functional interaction between the sodium pump and other synaptic proteins. Superresolution microscopy has thus opened up a new perspective to elucidate the nature of the physiological function, regulation and signaling role of Na^+^,K^+^-ATPase from its topological distribution in dendritic spines.

## Background

The Na^+^,K^+^-ATPase (NKA or sodium pump), is an integral plasma membrane protein complex responsible for the active transport of Na^+ ^and K^+ ^ions across the plasma membrane in almost all animal cells [1]. The sodium pump provides the electrochemical gradients for sodium and potassium that are essential for electrical excitability, secondary uptake and extrusion of ions, nutrients and neurotransmitters [2]. The sodium pumps role in mice behavioral defects has also been shown [3]. Studies have further indicated that the sodium pump may play a more dynamic role in neurons than what was previously believed. Recently, it was shown in a study on Drosophila neurons that the sodium pump mediates an after-hyperpolarization, which may interact with K^+ ^conductance, to provide a cellular memory of previous activity in the neuron [4].

The overall structural form of NKA appears as a heterotrimeric αβγ protein complex. The alpha subunit is the catalytic subunit and the main enzymatic properties of NKA are dependent of this isoform. It contains ten trans-membrane segments and both the N- and C-termini are intracellular [5]. The beta subunit contains a single membrane-anchoring helix and is essential for the delivery and appropriate insertion of the alpha subunit into the plasma membrane. The gamma subunit belongs to the polypeptide FXYD family and regulates the activity of the sodium pump in a tissue- and isoform-specific manner.

Two isoforms of the α-subunit are expressed in neurons, the ubiquitous α1 and the neuron specific α3 subunit [6,7]. It has also been shown that the α3 isoform has a lower sodium affinity and a higher affinity to extracellular potassium than the α1 isoform which suggests that the α3 isoform plays an important role in the excitatory synapse. The relatively low sodium affinity would endow the α3 isoform with a large reserve capacity for sodium and allow it to accommodate the large influxes of Na^+ ^that occur during repeated action potentials. The high potassium affinity would allow the α3 isoform to continue to function even when potassium is depleted due to pump mediated K^+ ^clearance [8].

Even though tissue and cell specific studies of the distribution of different α-isoforms have been done during the last decades [2,9], there is as yet little knowledge about the subcellular localization of NKA α-subunits in the brain. In this study we thus applied stimulated emission depletion microscopy (STED) to assess whether the α3 isoform is expressed in excitatory synapses located in spines. This novel microscopy technique, which gives nanoscale resolution, revealed that the α3 isoform was compartmentalized and clustered within dendritic spine structures. The anatomical finding was supported by biochemical studies, showing an interaction between neuronal NKA and the synaptic scaffolding protein PSD-95.

## Results

### Biochemical assays

**Figure 1:**
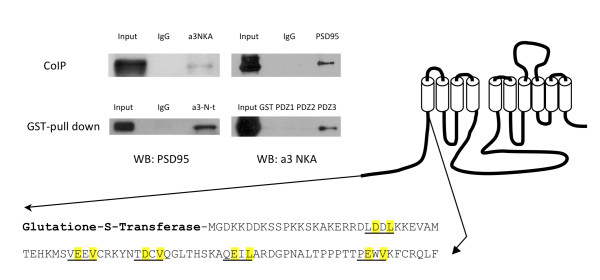
**Co-immunoprecipitation and GST pull down assay of PSD-95 and α3 NKA**. Co-immunoprecipitation (CoIP) shows interaction between PSD-95 and α3 NKA. GST pull down assays shows interaction of PSD-95 with the α3 NKA N-terminus and a specific PDZ3 interaction.

We first tested the possibility that the neuron specific α3 NKA is expressed in spines, using different biochemical methods. We found that the α3 isoform coimmunoprecipitated (CoIP) with the synaptic scaffolding protein PSD-95, a wellknown synaptic marker, typically located in the head of the spines in excitatory synapses [10]. Figure 1 shows Western blot images displaying this interaction, where the co-immunoprecipitation of the α3 NKA/PSD-95 complex was performed in five separate experiments using the α3 antibody and in three separate experiments using the PSD-95 antibody. To further confirm this interaction, we used glutathione-S-transferase (GST) fused peptides and the GST pull down technique (cf. Figure 1). It is well known that the N-terminus of the α-subunit of NKA can bind and interact with other proteins [11]. We thus generated a GST fused peptide corresponding to the Ntail of α3 NKA. This GST-fused N-tail of α3 NKA was found to pull down PSD-95. The PSD-95 protein contains several domains capable to bind with other proteins including three PDZ domains (UniProtKB/Swiss-Prot database entry P31016) also known as PDZ1, PDZ2 and PDZ3. By scanning the primary structure of rat α3 NKA against known protein motifs http://elm.eu.org we predicted five PDZ3 binding domains (LD23DL, VE39EV, TD48CV, QE60IL and PE78WV) in the N-tail of α3 in concordance with the consensus motif x[DE]x[IVL]. To find whether one or more of the three PDZ domains of PSD-95 will binds with rat α3 NKA, we produced soluble GST fused peptides corresponding to each of the three PDZ domains of rat PSD-95. It was found that the GST-fused PDZ3 domain effectively pulls down α3 NKA from the rat striatal lysate (cf. Figure 1). GST-pull down of PSD-95 using α3 NKA N-terminus was performed in five separate experiments. The pull down of α3 NKA with PSD-95 PDZ domains was performed in three separate experiments.

### STED microscopy

**Figure 2:**
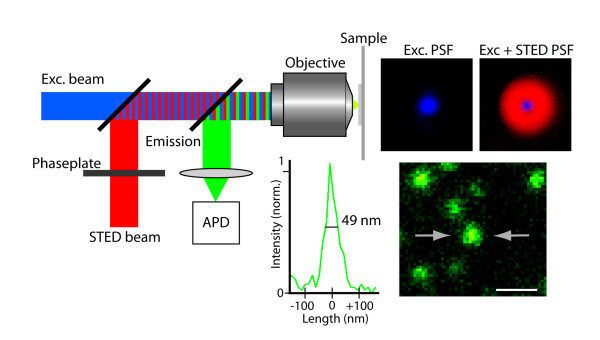
**Principle of STED microscopy**. The red depletion beam is phase-modulated to form a focal doughnut shown in the top right panel. Superimposition of this STED focus onto the diffraction limited excitation focus, shown in the adjacent panel, sharpens the effective fluorescence spot, which allows nanoscale imaging. The lower panels show well discernable fluorescent beads (diameter 40 nm) and a line profile across one of them with FWHM-value below 50 nm. Scale bar: 200 nm

The localization of α3 NKA subunits in dendritic spines was then studied in cultured neurons, derived from E18.5 day rat striatum (three separate cultures from three embryos of different litters were used). Due to the small dimensions of spines and the diffraction limit of light, it would not be possible to resolve the distribution of a protein within a single spine with classical fluorescence microscopy techniques. To overcome this inherent problem of limited resolution, we used the diffraction unlimited STED microscopy technique [12]. This superresolution technique shrinks a fluorescently activated spot by depleting the fluorescent state in a doughnut-shaped STED area, superimposed onto the excitation (cf. Figure 2). With sufficiently intense powers, the imaged fluorescent spot in a STED microscope can basically be scaled down to molecular sizes. By scanning the subdiffraction spot across the sample, in the same manner as in conventional confocal microscopy, fluorescence images with nanoscale resolution can thus be generated.

In our setup a super-continuum pulsed laser source for both excitation (λ_EXC _568 ± 5 nm) and STED (λ_STED _710 ± 10 nm) is utilized, which allows convenient preparation of synchronized laser pulses and selection of excitation and STED wavelengths. Its design has been described in detail before [13]. The resolving power of this set-up is shown in Figure 2, where fluorescent beads, with 40 nm diameter, have been used as calibration samples. Closely spaced beads are clearly discerned. Line profiles across individual beads have full-width-half-maximum (FWHM) values of 53 +/- 6 nm (n = 20 beads were measured). This value is however broadened by the physical size of the beads, indicating a FWHM resolution of around 40 nm.

**Figure 3:**
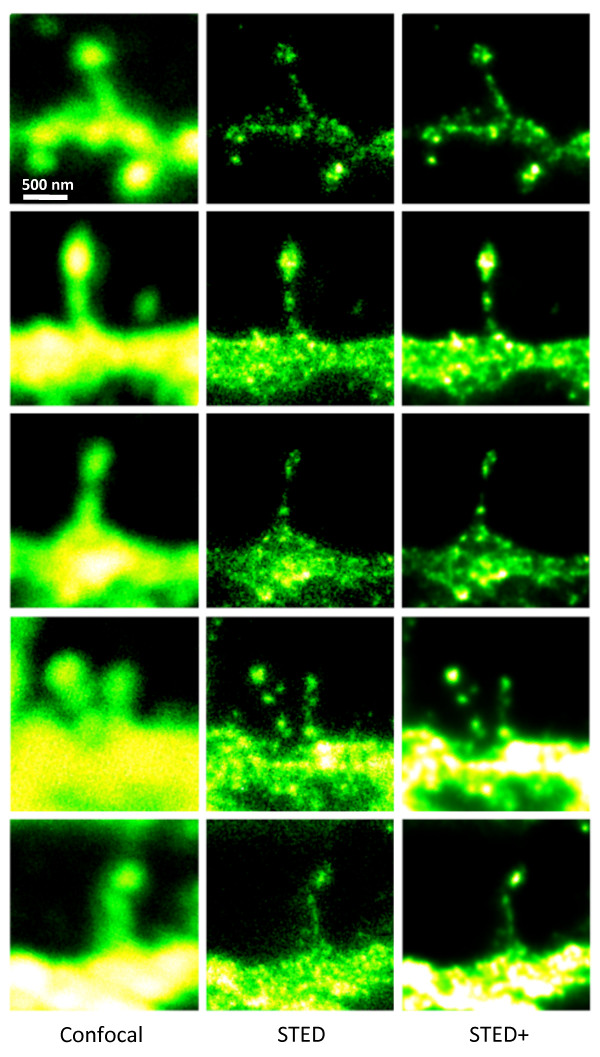
**STED microscopy dissecting the localization of Na^+^,K^+^-ATPase in cultured striatal neurons**. The 5 × 3 mosaic shows a comparision of the confocal (left) and STED images (middle) of the Alexa-594 immunolabelled α3 NKA in dendritic spines. Postprocessing of the raw STED data by a Richardson-Lucy deconvolution further enhances the details as shown in the right row of images. Scale bar: 500 nm

The use of STED microscopy made it possible to visualize the spine localization of NKA in unprecedented detail (n = 20 spines were analyzed). Figure 3 shows spineneck widths having FWHM-values of 82 +/-7 nm. Neuronal α3 NKA subunit was fluorescently immunolabeled with Alexa-594, a dye well suited for STED microscopy [14]. The STED images also revealed discernible pools of NKA complexes both in heads and necks of spines and also within the connecting dendritic structures. Notably, there are areas in the necks where there appears to be empty patches (cf. Figure 3A-E). Parts of the spine-necks are thus filled, but just below the head and/or above the shaft there are zones without α3 NKA subunits. Similar topological variations are also seen in the heads. Spine-heads viewed from the side, tilted, as well as from above (cf. Figure 3A-E), reveals a modulated distribution of α3 NKA complexes. The spatial size of these individual pools have FWHM-values of 58 +/- 11 nm, which physically limits the number of sodium pumps (size 65 × 75 × 150 Å) in a head-complex pool to contain not more than 20-30 subunits, assuming a mean density of 4-5 PSD-95 molecules per 1000 nm^2 ^and only one single sodium pump per scaffolding protein [15]. Steric hindrance by the relatively large antibody complexes may influence the estimate of NKA per nanocluster. It may on one hand give an overestimate if the antibodies physically increase the size of the imaged fluorescence cluster. On the other hand if quantification is based on the fluorescence intensity it may give an underestimate.

To compare the performance of STED imaging to conventional high resolution confocal microscopy, we sequentially acquired STED and confocal images of dendritic spines. The localization of α3 NKA in the STED images appears strikingly different from the localization recorded in the confocal reference images. In the confocal images the α3 NKA distribution appears as an unresolved continuous structure with an almost homogenous expression in both spine-heads and spine-necks. (cf. Figure 3A-E). Thus it is obvious that no topological variations of the NKA distribution in spines can be visualized with conventional optical microscopy techniques.

## Discussion

The composition, turnover and localization of proteins in spines play a major role for brain plasticity [16]. Superresolution fluorescence STED microscopy has previously been used to visualize immunolabeled presynaptic proteins [17-19], neuromuscular junctions [20], and to record the nanoscale morphological dimensions of YFP-labelled dendritic spines [21]. To the best of our knowledge, the performed work in the present study is the first, where the nanoscale distribution of a protein in spines has been visualized with STED microscopy. The resulting STED images showed that neuronal NKA is located in discernible pools in the spine-heads and spine-necks as well as within the connecting dendritic structures. Such detailed localization of α3 NKA could not be dissected with conventional optical microscopy. This has limited previous investigation to apply electron microscopy for ultrastructual localization of NKA [22].

The discontinuous distribution of NKA in spines will have implications for the structural and functional interaction between NKA and other synaptic proteins and for the generation of local sodium gradients within the spine. For example, the recent work of Pulver and Griffith [4] on Drosophila neurons have suggested that NKA mediated Na+ efflux after an action potential plays a more dynamic role than what was previously assumed. It was shown that the bursts of action potentials were followed by a pump-mediated after-hyperpolarization event that was able to function as an integrator of spike number for multiple seconds. The function of such a process should require a well-balanced spatial relationship between the sodium influx channels and the sodium pump, and it will be an important future task to record with superresolution microscopy the dynamic interaction between, for example, α3 NKA and AMPA receptors in spines.

The localization of subpopulations of NKA in specific spine regions might also have an impact on pump activity. The activity of NKA can be regulated by ligand binding and by phosphorylation/dephosphorylation processes. Recent studies have shown that the C-terminus tail of agrin is a NKA ligand and inhibitor of pump function [23]. This fragment is specifically cleaved by neurotrypsin in the synapse. Uncleaved agrin is additionally involved in acetylcholine receptor pre-synaptic accumulation. A link and interplay between signaling and pumping is therefore established that control the dynamic regulation of sodium. NKA thus play the role of a transducer of extracellular signals to the cytoplasmic space [24]. Also protein kinase A (PKA) mediated phosphorylation of NKA [25], which can be mediated by the dopamine D1 receptor (D1R), influence pump activity of NKA by a decrease in sodium affinity [26]. This indicates yet another coupling of the sodium pump to a neurotransmitter activated process. A future task is to apply dual-color and live-cell STED microscopy to elucidate the physiological implications of clustered and compartmentalized NKA distributions in different cell types, and with several interacting proteins.

## Conclusions

The dissected appearance of NKA free patches in the spine necks has important implications for the control of intracellular sodium concentration in the spine head. The intracellular space of the spine is often considered to function as a discreet chemical compartment [27], isolating the concentration dynamics of ions and intracellular messengers from the dendritic shaft and from neighboring spines. An elegant study by Rose and Konnerth [28], performed on hippocampal neurons, has shown that there are indeed large sodium gradients between spines and adjacent dendrites. Their finding that these gradients are maintained over hundreds of milliseconds, suggests that there is a diffusion barrier between the spine-head and the shaft, and where the spine-neck constitute the physical structure of this barrier. The spine-head accumulation of α3 isoforms, with its relatively low sodium affinity, should thus contribute to maintain a higher sodium concentration in the semi-isolated spine. Partial lack of NKA expression in the neck should contribute to the maintenance of a relatively high sodium concentration in spines as compared to the adjacent dendritic shaft. The physiological role of the relatively high intracellular sodium concentration in spines remains to be clarified, but it is likely that it will contribute to the modulation of the sodium sensitive K^+ ^channels expressed postsynaptically in many regions of the brain, including the hippocampus [29].

Taken together, STED microscopy has now opened a new avenue for studying the spatial interaction between Na^+^,K+-ATPase and modulators of its activity. Provided with much sharper nanoscale resolving eyes, an even deeper and more thorough analysis of this and several other receptor-protein interactions in spines is thus doable.

## Methods

### Cell culture

Cultures of striatal neurons were prepared from embryonic day 18.5 old Sprauge dawley rat embryos. Striatum was dissected, incubated for 10 minutes at 37°C in Hank's balanced salt solution (Gibco Invitrogen) containing 20 mM HEPES (Sigma) and 0.25% Trypsin (Gibco Invitrogen) and dissociated in MEM (Gibco Invitrogen) by mechanical triturating using a fire polished Pasteur pipette. The cells were plated at a density of 2 × 10^5 ^cells on round 28 mm, No. 1.0, poly-ornithine (Gibco Invitrogen, 80 μg/mL over night at 37°C) coated coverslips and incubated for three hours in MEM media containing 10% Horse serum, 2 mM L-Glutamine and 1 mM NaPyruvate. The cells where then cultured in Neurobasal media (Gibco Invitrogen) containing 1× B27 (Gibco Invitrogen) and 2 mM L-Glutamine. Cells were maintained in culture for two weeks before experiments and culture media was changed once a week.

### Immunostaining

Cells were fixed for 15 min in ice cold methanol, permeabilized with 0.1% triton and blocked with 7% normal goat serum and then incubated overnight at 4°C with primary antibody diluted 1:1000 in PBS containing 3% normal goat serum and 0.1% Triton X-100. The primary antibody was mouse monoclonal anti-α3 Na ^+^,K^+^-ATPase (Affinity bioreagents). Following incubation the cells were washed in PBS, 0.1% triton and incubated at room temperature for two hours with fluorescent secondary antibody, goat-anti-mouse Alexa-594 (Molecular Probes Inc.) diluted 1:500 in PBS with 3% serum and 0.1% Triton X-100. Following secondary incubation cells were washed again, PBS, 0.1% Triton X-100 and mounted using Immu-mount (Thermo scientific).

### DNA constructs

The full length intracellular N-tail of α3 NKA was obtained from rat brain total RNA as RT-PCR fragment with additional CACC leading sequence and a stop codon introduced after the end of the particular domain. cDNA were then cloned into pENTR-D vector with the following exchange into pDEST15 expression vector by Gateway TOPO cloning system (Invitrogen, USA). The structure of GST-fusion plasmids was confirmed by sequencing with BigDye v.3.1 (Applied Biosystems, USA).

### Antibodies

In both imaging and biochemical assays the well characterized mouse monoclonal anti-α3 Na ^+^,K^+^-ATPase (Affinity bioreagents, MA3-915) was used [30]. For Western blotting and immunoprecipitation, the following antibodies were used: mouse monoclonal anti-PSD-95 (Abcam, Cambridge, UK) and as control mouse IgG (Sigma Aldrich, St.Louis, MO, USA). The used antibodies show high specificity with single bands in Western blot experiments and very low unspecific labelling in imaging experiments.

### Immunoprecipitation

Striatum from 20 days old rats was homogenized in the RIPA buffer containing 50 mM Tris HCl (pH 7,4), 150 mM NaCl, 2 mM EDTA, 1 mM phenylmethylsulfonyl fluoride, 0.25% sodium deoxycholate, 1% Triton X-100 and protease inhibitors (Pierce, Rockford, IL, USA) and centrifuged 30 min at 9000 g. Supernatant protein concentration was measured. 1 ml of the striatal lysate (300 μg of total protein) was pre-cleared with 60 μl of protein G Sepharose beads (GE Healthcare Bio-Sciences AB, Uppsala, Sweden) 1 hour at 4°C. Lysate was then incubated with 3 μg of α3 Na^+^,K^+^-ATPase, PSD-95, or mouse IgG antibodies for 2 hours at 4°C. 30 μl of protein G Sepharose beads was added and incubation continued over night at 4°C.

The samples were washed 5 times with RIPA buffer and the proteins were eluted with 50 μl of 2× Laemmli buffer for 15 min at 65°C. Samples were then subjected to SDS-PAGE for Western blotting.

### GST affinity pull-down

Expression and purification of the GST-fused proteins were performed as described previously [31]. Rat striatal lysate (300 μg of total protein) was prepared in the RIPA buffer and added to the beads in a volume of 1 ml and incubated for 4 hours at 4°C. The beads were then washed four times with RIPA buffer. Proteins were eluted with 50 μl of 2× Laemmli buffer for 15 min at 65°C. Samples were then subjected to SDS-PAGE for Western blotting, using antibodies for: PSD-95 (1:1000) and α3 NKA (1:1000). The experimental procedure was repeated at a minimum of three times.

### STED microscopy

The stimulated emission depletion microscope has been described in detail before [13]. Our setup has the following minor differences: The excitation and STED beams are coupled together using a dichroic mirror (z690SPRDC, Chroma Technology Corp., Bellow Falls, US) before being sent into the microscope objective; The fluorescence from the labeled sample is collected back through the objective and separated from the excitation and the STED beams by a customized dichroic mirror (Laseroptik, Garbsen, Germany); The sample is placed on a 3 D scanning piezo stage coupled to a closed loop controller unit (MAX311/M and BPC203, Thorlabs Sweden AB, Göteborg, Sweden) offering a positional resolution of 5 nm. Typically images between 7 μm × 7 μm to 15 μm × 15 μm were acquired with a pixel size of 10 - 30 nm and a pixel dwell time of 0.5-1 ms. The average excitation powers applied were 0.5-1.1 μW and the applied STED powers were 2.6-3.0 mW.

## Authors' contributions

HAB constructed the STED microscope, analyzed the imaging data and drafted the manuscript. DR constructed the microscope, did the imaging and helped with analyzis of data. LS did the cell culture and immunostaining and participated in experimental design. ZS designed and performed the GST immunoprecipitation and pull down assays. JW initiated the STED microscope construction and helped in data analysis. AB made the DNA constructs and helped in GST pull down assays and immunoprecipitation studies. AA and HJB conceived the study, participated in its design and coordination and drafted the manuscript. All authors read and approved the final manuscript.
